# Systemic complement levels in patients with age-related macular degeneration carrying rare or low-frequency variants in the *CFH* gene

**DOI:** 10.1093/hmg/ddab256

**Published:** 2021-09-11

**Authors:** Sarah de Jong, Anita de Breuk, Elena B Volokhina, Bjorn Bakker, Alejandro Garanto, Sascha Fauser, Suresh Katti, Carel B Hoyng, Yara T E Lechanteur, Lambert P van den Heuvel, Anneke I den Hollander

**Affiliations:** Department of Ophthalmology, Donders Institute for Brain, Cognition and Behavior, Radboud University Medical Center, 6525 GA Nijmegen, The Netherlands; Department of Ophthalmology, Donders Institute for Brain, Cognition and Behavior, Radboud University Medical Center, 6525 GA Nijmegen, The Netherlands; Amalia Children’s Hospital, Radboud University Medical Center, 6525 GA Nijmegen, The Netherlands; Radboud Institute for Molecular Life Sciences, Radboud University Medical Center, 6525 GA Nijmegen, The Netherlands; Department of Laboratory Medicine, Radboud University Medical Center, 6525 GA Nijmegen, The Netherlands; Department of Ophthalmology, Donders Institute for Brain, Cognition and Behavior, Radboud University Medical Center, 6525 GA Nijmegen, The Netherlands; Amalia Children’s Hospital, Radboud University Medical Center, 6525 GA Nijmegen, The Netherlands; Radboud Institute for Molecular Life Sciences, Radboud University Medical Center, 6525 GA Nijmegen, The Netherlands; Department of Laboratory Medicine, Radboud University Medical Center, 6525 GA Nijmegen, The Netherlands; Department of Human Genetics, Radboud University Medical Center, 6525 GA Nijmegen, The Netherlands; Department of Ophthalmology, University Hospital of Cologne, 50937 Cologne, Germany; F. Hoffmann-La Roche AG, 4070 Basel, Switzerland; Gemini Therapeutics Inc., Cambridge, MA 02139, USA; Department of Ophthalmology, Donders Institute for Brain, Cognition and Behavior, Radboud University Medical Center, 6525 GA Nijmegen, The Netherlands; Department of Ophthalmology, Donders Institute for Brain, Cognition and Behavior, Radboud University Medical Center, 6525 GA Nijmegen, The Netherlands; Amalia Children’s Hospital, Radboud University Medical Center, 6525 GA Nijmegen, The Netherlands; Radboud Institute for Molecular Life Sciences, Radboud University Medical Center, 6525 GA Nijmegen, The Netherlands; Department of Laboratory Medicine, Radboud University Medical Center, 6525 GA Nijmegen, The Netherlands; Department of Ophthalmology, Donders Institute for Brain, Cognition and Behavior, Radboud University Medical Center, 6525 GA Nijmegen, The Netherlands; Department of Human Genetics, Radboud University Medical Center, 6525 GA Nijmegen, The Netherlands

## Abstract

Age-related macular degeneration (AMD) is a major cause of vision loss among the elderly in the Western world. Genetic variants in the complement factor H (*CFH*) gene are associated with AMD, but the functional consequences of many of these variants are currently unknown. In this study, we aimed to determine the effect of 64 rare and low-frequency variants in the *CFH* gene on systemic levels of factor H (FH) and complement activation marker C3bBbP using plasma samples of 252 carriers and 159 non-carriers. Individuals carrying a heterozygous nonsense, frameshift or missense variant in *CFH* presented with significantly decreased FH levels and significantly increased C3bBbP levels in plasma compared to non-carrier controls. FH and C3bBbP plasma levels were relatively stable over time in samples collected during follow-up visits. Decreased FH and increased C3bBbP concentrations were observed in carriers compared to non-carriers of *CFH* variants among different AMD stages, with the exception of C3bBbP levels in advanced AMD stages, which were equally high in carriers and non-carriers. In AMD families, FH levels were decreased in carriers compared to non-carriers, but C3bBbP levels did not differ. Rare variants in the *CFH* gene can lead to reduced FH levels or reduced FH function as measured by increased C3bBbP levels. The effects of individual variants in the *CFH* gene reported in this study will improve the interpretation of rare and low-frequency variants observed in AMD patients in clinical practice.

## Graphical Abstract

**Graphical Abstract:**
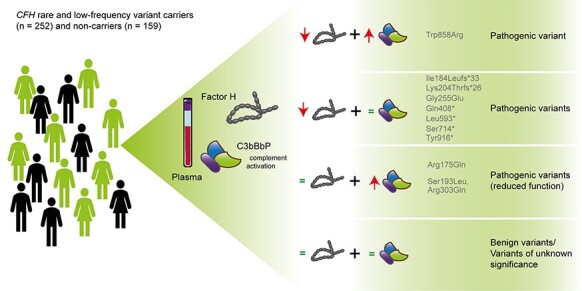

## Introduction

Age-related macular degeneration (AMD) is a major cause of vision loss among elderly in the Western world. The number of affected individuals is expected to grow in the coming years in line with an increased ageing of the population (1). AMD is characterized by accumulation of extracellular deposits, called drusen, between the basal lamina of the retinal pigment epithelium (RPE) and the inner collagenous layer of Bruch’s membrane (2) and by pigmentary changes of the RPE. During disease progression, drusen tend to expand and coalesce, eventually leading to advanced AMD. Advanced AMD is divided into two forms: neovascular AMD and geographic atrophy. Neovascular AMD is defined by the formation of aberrant blood vessels, invading the retina. These fragile vessels tend to leak, thereby leading to rapid vision loss. Geographic atrophy is characterized by gradually progressing atrophy of the outer retinal tissue, RPE and choriocapillaris, resulting in progressive and irreversible vision loss (3). Neovascular AMD can be treated with vascular endothelial growth factor inhibitors, but for geographic atrophy no cure exists as of today (4). AMD has a multifactorial etiology, and many factors modifying AMD risk have been identified, including ageing, life style (smoking, diet, antioxidants) and genetic predisposition (3). A strong link between genetic variation in the complement system and AMD has been established (5).

The complement system is an ancient part of innate immunity and plays an important role in the first line defense against pathogens. The complement system can be activated via three different pathways: the classical pathway, the lectin pathway and the alternative pathway. The alternative pathway is continuously activated at a low level by tick-over of C3 into C3(H_2_O), which resembles the activation product C3b. C3(H_2_O) and C3b bind Factor B (FB). Bound FB is cleaved by Factor D (FD), resulting in the formation of C3 convertase C3bBb, which is stabilized by additional binding of properdin (C3bBbP). The formed C3bBbP complex cleaves C3 into the anaphylatoxin C3a and C3b; the newly formed C3b fragments on one hand generate new C3 convertases (amplification-loop), but on the other hand bind to the C3bBbP complex resulting in C5 convertase formation (C3bBbPC3b). The C5 convertase cleaves C5 into the anaphylatoxin C5a and C5b. C5b initiates sequential binding of the complement components C6, C7, C8 and multiple C9 molecules and formation of the C5b-9 membrane attack complex (MAC). Successful MAC assembly on a target membrane can lead to lysis of the target cell. Other effects of complement activation include tagging target cells for opsonization and triggering clearance of non-host cells through non-inflammatory mechanisms or inflammatory response due to anaphylatoxin generation (6).

**Figure 1:**
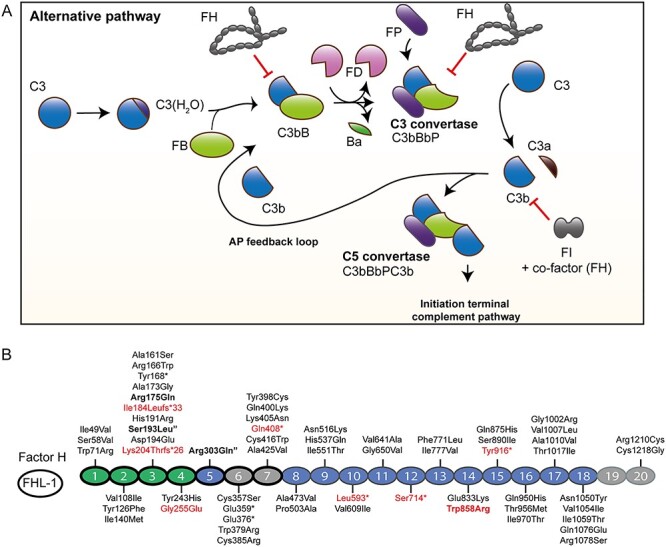
Overview of the alternative pathway and the distribution of variants in FH. (**A**) An overview of the alternative pathway of the complement system is given. C3 shows spontaneous tick-over to C3(H_2_O), which resembles the activation product C3b. FB binds to C3(H_2_O), and is then cleaved by FD, resulting in the formation of active C3 convertase (C3bBb). Additional binding of Properdin (FP) stabilizes the C3 convertase (C3bBbP). The C3 convertase cleaves C3 to C3a and C3b, thereby enabling generation of more C3b. Additional binding of C3b to the C3 convertase results in formation of C5 convertase (C3bBbPC3b), which cleaves C5. Cleavage of C5 results in initiation of MAC formation. The inhibitors FH and FI prevent excessive complement activation and thereby damage of host tissue. FI cleaves C3b in the presence of a co-factor. FH acts as a co-factor for FI-mediated C3b degradation, competes with FB binding to C3b and accelerates the decay of the C3 and C5 convertases. (**B**) Schematic representation of the FH protein. Variants are shown for each CCP of FH. Two variants not located in structures of the mature protein (p.Leu3Val located in the signal peptide, and the splice site deletion c.1697-17_1697-8del) are not shown. The functional domains of FH are indicated in green and light gray, and the CCPs present in FHL-1 indicated with a black outline. FHL-1 contains additional amino acids (Ser, Phe, Thr and Leu) after CCP7, which are not depicted in the figure. CCPs1–4 are essential for decay-accelerating and co-factor activity (green) and are also involved in C3b binding, and CCP19–20 contain also a C3b binding site. CCP6–7 and CCP19–20 contain binding sites for glycosaminoglycans (light gray). Variants with significantly reduced FH plasma levels are indicated in red, and variants with significantly increased C3bBbP levels in plasma are indicated in bold. The two variants p.Ser193Leu and p.Arg303Gln were only present in combination, and are marked with a (“).

In order to prevent host tissue damage tight control of complement activation is essential, and approximately half of the complement components function as regulators of complement activation. A major complement regulator is Factor H (FH). FH consists of 20 complement control protein (CCP) domains and acts as a co-factor for Factor I (FI)-mediated C3b degradation but also competes with binding of FB to C3b and accelerates decay of the C3 convertase (decay accelerating activity), thereby preventing further C3b generation and activation of the amplification loop (Fig. 1A).

The smaller, alternative splice form of FH, Factor H-like 1 (FHL-1), was identified as a form present in the retina since, in contrast to FH, it can pass Bruch’s membrane and thereby facilitate local regulation of complement activation (Fig. 1B) (7).

Genetic variants in the complement factor H (*CFH*) locus are strongly associated with AMD. A genome-wide association study identified eight variants that are independently associated with AMD (5). The lead variants include one rare missense variant c.3628C>T (p.Arg1210Cys), which exerts a very strong effect on AMD (odds ratio ~20). The remaining variants are synonymous or non-coding, of which one, rs570618, is in high linkage disequilibrium (LD) with the c.1204T>C (p.Tyr402His) variant in *CFH*. The p.Arg1210Cys variant affects the binding of FH to human serum albumin (8) and thereby its availability as a regulator, while the p.Tyr402His variant shows altered binding to extracellular components like heparan sulfate present in Bruch’s membrane (9). In both cases, the altered availability of FH results in reduced function of the protein. AMD patients also carry a significantly higher burden of rare variants in *CFH* compared to control individuals (5). Overall, more than 100 different rare variants have been detected in AMD case–control and family studies (10). However, for the majority of these variants, the functional effects and potential contribution to AMD risk are not known (11).

The aim of this study was to determine the effects of rare and low-frequency variants, defined by a minor allele frequency of <0.01 or between 0.01 and 0.05, respectively, in the *CFH* gene by measuring systemic levels of FH and C3bBbP, as a complement activation marker, in a large cohort of carriers of rare and low-frequency variants in *CFH*.

## Results

### Cohort description

In total, 168 unrelated carriers of rare and low-frequency *CFH* variants [median age 70.7 years (IQR 66.4–78.1 years), 64% female], 105 unrelated non-carriers [median age 75.9 years (IQR 73.5–79.5 years), 61% female], 84 family members carrying *CFH* variants [median age 62.8 years (IQR 49.6–71.8 years), 57% female] and 54 non-carrier family members [median age 58.8 years (IQR 42.2–70.5 years), 54% female] were included for analysis (Table 1). It should be noted that, except between carriers and non-carriers of the family cohort, the median age between all groups differs significantly (*P* < 0.001, Kruskal–Wallis test with Dunn’s multiple comparison test). In total, 63 different rare variants and one low-frequency variant [c.3148A>T (p.Asn1050Tyr)] were identified in 252 individuals. Of the 252 individuals, 16 (6.3%) individuals carry heterozygous nonsense or frameshift mutations, 24 (9.5%) individuals carry heterozygous missense variants with a predicted damaging effect [Combined Annotation-Dependent Depletion (CADD) score ≥20], 186 (73.8%) individuals carry heterozygous missense variants predicted to be benign (CADD score <20), 1 (0.4%) individual carries a splice site variant and 25 (9.9%) individuals carry two or more *CFH* variants (Supplementary Material, Table S1). The identified variants are distributed throughout the entire FH protein (Fig. 1B).

**Table 1:** Characteristics of included individuals

	Family cohort		Case–control cohort	
	*CFH* carriers	*CFH* non-carriers	*CFH* carriers	*CFH* non-carriers
Age, median (IQR)	62.8 (49.6–71.8)^*^	58.8 (42.2–70.5)^*^	70.7 (66.4–78.1)^*^	75.9 (73.5–79.5)^*^
Gender, No. (%)				
Male	36 (42.9)	25 (46.3)	61 (36.3)	41 (39.0)
Female	48 (57.1)	29 (53.7)	107 (63.7)	64 (61.0)
Phenotype, No. (%)				
No AMD	35 (41.7)	41 (75.9)	85 (50.6)	53 (50.5)
Early/intermediate AMD	25 (29.8)	10 (18.5)	38 (22.6)	25 (23.8)
Advanced AMD	24 (28.6)	3 (5.6)	45 (26.8)	27 (25.7)
Total No.	84	54	168	105

^*^Except between carriers and non-carriers of the family cohort, the median age between all groups differs significantly (*P* < 0.001, Kruskal–Wallis test with Dunn’s multiple comparison test).

In 25 individuals, multiple low-frequency variants were identified; for 11 of these individuals, genotype data of family members were available, enabling segregation analysis to determine whether the variants are on the same allele (Supplementary Material, Fig. S1 and Fig. 6D). The variants c.481G>T (p.Ala161Ser) and c.2850G>T (p.Gln950His) were identified in family 23 and are not co-inherited on the same allele (Supplementary Material, Fig. S1a). The variant combination c.578C>T; 908G>A (p.Ser193Leu; Arg303Gln) was identified in three families (families 24, 25 and 26). In family 25, only one carrier was identified. However, in families 24 and 26, both variants are consistently co-inherited; therefore, it is possible that the carrier in family 25 (II.1) also carries both variants on the same allele (Supplementary Material, Fig. S1b–d). In one carrier, the variants c.2850G>T (p.Gln950His) and c.3148A>T (p.Asn1050Tyr) were identified (family 28, III.1). Both variants also occur individually in other family members and therefore are not on the same allele (Fig. 6D). The variant combination c.2669G>T; 3019G>C (p.Ser890Ile; Val1007Leu) was identified in four unrelated individuals, and these variants are in high LD (*D*′ = 1.0, *R*^2^ = 1.0) (https://ldlink.nci.nih.gov/?tab=ldmatrix). Therefore, and in line with previously reported haplotype analysis (12), these variants may be co-inherited on the same allele. The variant combination c.2669G>T; 3176C>T (p.Ser890Ile; Ile1059Thr) was identified in one sporadic case, and these variants are also in high LD (*D*′ = 1.0, *R*^2^ = 0.028) when looking at all populations, indicating the two variants might be on the same allele. One individual of multiracial ethnicity presented with a combination of the variants c.[2669G>T; 3019G>C; 3176C>T]; [3019G>C; 3050C>T] (p.[Ser890Ile; Val1007Leu; Ile1059Thr]; [Val1007Ile; Thr1017Ile]). One individual carries the variant combination c.1418C>T(;)2850G>T (p.Ala473Val(;)Gln950His), and in multiple individuals, a combination of the low-frequency variant c.3148A>T (p.Asn1050Tyr) with a rare variant [c.1198C>A (p.Gln400Lys), c.2850G>T (p.Gln950His), c.3130A>G (p.Arg1044Gly) and c.211T>A (p.Trp71Arg)] was identified. For these seven individuals, no family members were available for segregation analysis; therefore, we could not assess whether the variants are on the same allele or not.

The common variant c.1204T>C (p.Tyr402His) shows a strong association with AMD risk and affects both FHL-1 and FH, due to its location in CCP7. Therefore, the frequency of the variant rs570618, which is in LD with the p.Tyr402His variant, was analyzed in the non-carriers, carriers of a *CFH* variant in CCP1–7 and carriers of a *CFH* variant in CCP8–20 using chi-squared test (Supplementary Material, Table S2). Strikingly, when including all rare variant carriers, the allele frequencies differ significantly from the expected frequencies. However, the variants c.2850G>T (p.Gln950His) and c.3148A>T (p.Asn1050Tyr) are in high LD with the protective allele of the c.1204T>C (p.Tyr402His) variant (*D*′ = 1.0, *R*^2^ = 0.004 and *D*′ = 1.0, *R*^2^ = 0.008, respectively; https://ldlink.nci.nih.gov/?tab=ldmatrix). Therefore, the analysis was repeated excluding carriers of these two variants individually or in combination with another variant. When excluding carriers of the variants c.2850G>T (p.Gln950His) and c.3148A>T (p.Asn1050Tyr), the allele frequencies of rs570618 do not differ from the expected frequencies (Supplementary Material, Table S2).

### Plasma FH and C3bBbP plasma levels in carriers of rare and low-frequency CFH variants

**Figure 2:**
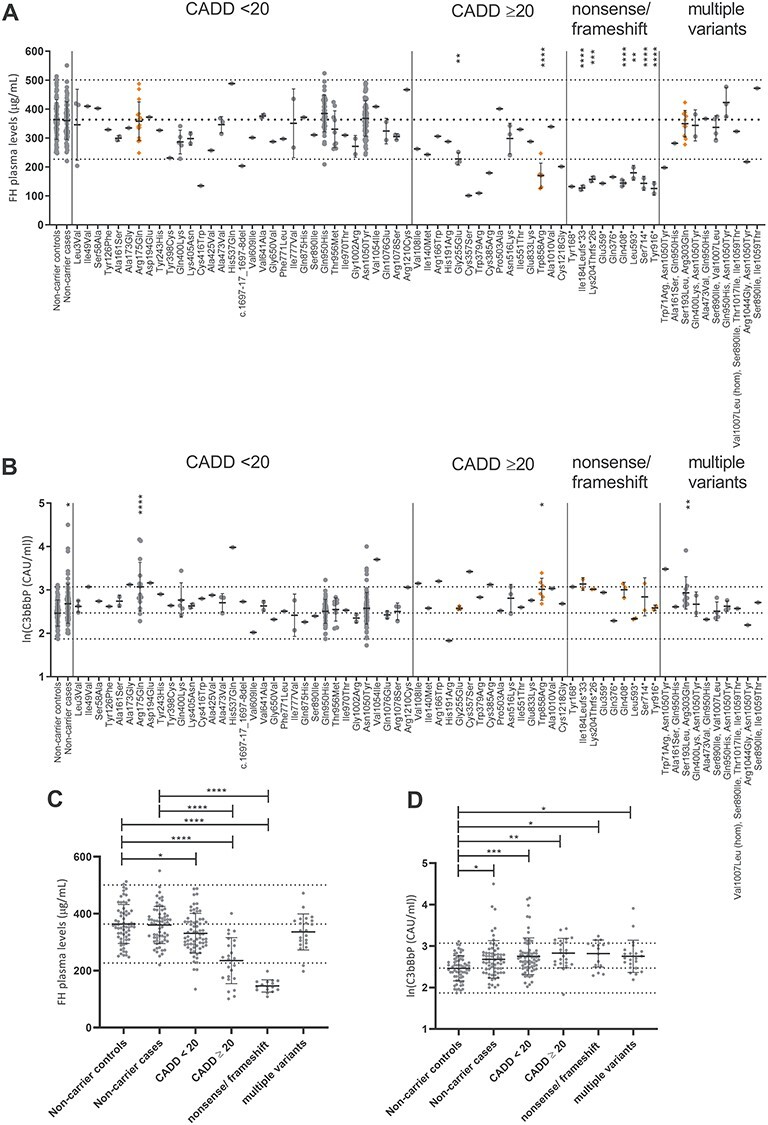
FH and C3bBbP plasma levels in *CFH* rare and low-frequency variant carriers. (**A**, **C**) FH and (**B**, **D**) ln(C3bBbP) plasma levels are shown in non-carrier controls, non-carrier AMD cases and *CFH* low-frequency variant carriers. Dotted lines indicate the mean ± 2 SD, in (A, C) 363.7 μg/ml ± 2^*^68.5 μg/ml FH and in (B, D) 2.47 ln(CAU/ml) ± 0.30 ln(CAU/ml) C3bBbP. Mean and SD are shown by error bars. (A, B) Missense variants are sorted based on CADD score with CADD = 20 as cut-off. Variant groups were compared with non-carrier controls using ordinary one-way ANOVA and Dunnett’s multiple comparison test, and significant *P*-values are indicated with ^*^<0.05, ^*^^*^<0.01, ^*^^*^^*^<0.001 and ^*^^*^^*^^*^<0.0001. Variants leading to significantly increased ln(C3bBbP) levels are indicated in orange in (A) and variants leading to significantly decreased FH levels are indicated in orange in (B). (C) FH and (D) C3bBbP plasma levels are shown for all individuals grouped based on presence of missense variants with a CADD score <20, missense variants with a CADD score ≥20, nonsense/frameshift variants or presence of multiple low-frequency variants and compared to non-carrier cases and non-carrier controls using ordinary one-way ANOVA with Sidak’s multiple comparison test, significant *P*-values are indicated with ^*^<0.05, ^*^^*^<0.01, ^*^^*^^*^<0.001 and ^*^^*^^*^^*^<0.0001.

For each plasma, sample FH and C3bBbP levels were determined by ELISA in technical duplicates, and the mean for each individual sample is shown in Figure 2. Carriers of the variants c.550delA (p.Ile184Leufs^*^33), c.610insCCAA (p.Lys204Thrfs^*^26), c.764G>A (p.Gly255Glu), c.1222C>T (p.Gln408^*^), c.1778T>A (p.Leu593^*^), c.2141C>G (p.Ser714^*^), c.2572T>A (p.Trp858Arg) and c.2748C>G (p.Tyr916^*^) showed significantly reduced FH levels in plasma compared to non-carrier controls. In addition, individuals carrying variants c.1248C>G (p.Cys416Trp), c.1697-17_1697-8del, c.1069T>A (p.Cys357Ser), c. 1135T>A (p.Trp379Arg), c.1153T>C (p.Cys385Arg), c.3652T>G (p.Cys1218Gly), c.504C>A (p.Tyr168^*^), c.1075G>T (p.Glu359^*^), and c.1126C>T (p.Gln376^*^) and two individuals with the variant combinations c.211T>A(;)3148A>T (p.Trp71Arg(;)Asn1050Tyr) and c.3130A>G(;)3148A>T (p.Arg1044 Gly(;)Asn1050Tyr), respectively, showed FH plasma levels below the normal range defined by the mean ± 2SD in non-carrier controls (363.7 μg/ml ± 2^*^68.5 μg/ml). For these variants, only one individual carrier was available; therefore, they could not be included in the statistical analysis (Fig. 2A).

The C3bBbP plasma levels showed a log-normal distribution and were therefore transformed with the natural log for further analysis. Individuals carrying the variants c.524G>A (p.Arg175Gln), c.2572T>A (Trp858Arg) and c.578C>T;908G>A (p.Ser193Leu;Arg303Gln), and non-carrier cases showed significantly increased C3bBbP levels compared to non-carrier controls. Individuals carrying the variants c.518C>G (p.Ala173Gly), c.582C>A (p.Asp194Glu), c.1611T>A (p.His537Gln), c.3160G>A (p.Val1054Ile), c.322G>A (p.Val108Ile), c.496C>T (p.Arg166Trp), c.1611T>A (p.Cys357Ser), c.1153T>C (p.Cys385Arg) and one individual with the variants c.211T>A(;)3148A>T (p.Trp71Arg(;)Asn 1050Tyr) showed C3bBbP plasma levels above the normal range determined by the non-carrier control mean ± 2 SD (2.47 ln(CAU/ml) ± 0.30 ln(CAU/ml)) (Fig. 2B). For 42 variants, plasma from only one individual was available; therefore, these were excluded from further statistical analysis.

**Figure 3:**
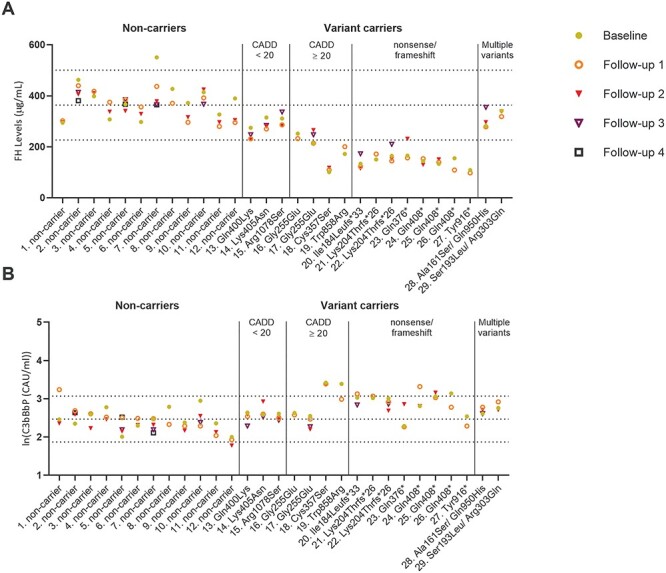
FH and C3bBbP plasma levels in follow-up samples of non-carrier AMD cases and AMD cases carrying rare *CFH* variants. (**A**) FH and (**B**) ln(C3bBbP) levels are shown in up to five samples from non-carrier AMD cases or AMD cases carrying rare *CFH* variants. Dotted lines indicate the mean ± 2 SD based on the non-carrier controls shown in Figure 2. Up to four follow-up, samples were collected in a time frame of 0.5–12 years after collection of the baseline sample. Details for each individual are given in Supplementary Material, Table S2. The different samples are indicated according to the following: baseline = yellow dot, follow-up 1 = orange circle, follow-up 2 = red triangle (filled), follow-up 3 = purple triangle (outline) and follow-up 4 = dark purple square (outline). Numbers 1–29 represent the different individuals as described in Supplementary Material, Table S2.

Next, individuals were grouped based on the type of variant they carried, resulting in four groups: variants with a CADD score <20, missense variants with a CADD score ≥20, nonsense and frameshift variants, and individuals with multiple variants. Individuals carrying the variants c.2850G>T (p.Gln950His) (*N* = 41) and c.3148>T (p.Asn1050Tyr) (*N* = 77) were excluded from this analysis, in order to prevent that the effects of other variants are masked by the large number of carriers and the protective effect of these variants (5,10). Individuals carrying a variant with a CADD ≥20 and individuals carrying a nonsense or frameshift variant showed significantly reduced FH plasma levels compared to non-carrier controls and non-carrier cases, and individuals with a variant with CADD <20 showed significantly decreased FH plasma levels compared to non-carrier controls (Fig. 2C). C3bBbP plasma levels were significantly elevated in non-carrier cases, carriers of a variant with a CADD <20, with a CADD ≥20, in carriers with a nonsense or frameshift variant and in carriers of multiple variants compared to non-carrier controls (Fig. 2D). The alternative splice form of FH, FHL-1, contains the first seven CCPs plus four unique amino acids on the C-terminus. Consequently, variants in the first seven CCPs affect both products, while variants located in CCP8–20 will only affect full-length FH. When stratifying carriers based on whether the variant is located in CCP1–7 or in CCP8–20, both groups show equally reduced FH plasma levels compared to non-carrier controls and non-carrier cases (Supplementary Material, Fig. S2a). When comparing the effect on C3bBbP plasma levels, however, carriers of rare *CFH* variants located in CCP1–7 show significantly elevated C3bBbP plasma levels compared to the non-carrier controls, non-carrier cases and carriers of rare CFH variants located in CCP8–20, whereas C3bBbP levels in carriers of rare *CFH* variants located in CCP8–20 were not elevated compared to the non-carrier controls and non-carrier cases (Supplementary Material, Fig. S2b).

### Plasma FH and C3bBbP plasma levels in follow-up samples

To evaluate whether FH and C3bBbP levels were stable over time, we selected plasma samples collected at up to five visits from 29 AMD patients (12 non-carriers, 17 carriers). The follow-up visits took place between 0.5 and 11.6 years after the baseline visit (Supplementary Material, Table S3). FH and C3bBbP plasma levels were relatively stable in the same individual over time, with only three (10%) cases in which FH plasma levels were not consistently within the normal range (individuals 7, 17 and 23), and seven (24%) cases in which C3bBbP plasma levels were not consistently inside or outside the normal range (individuals 1, 12, 19, 20, 24, 25 and 26) (Fig. 3). The FH measurements in individuals carrying the c.1069T>A (p.Cys357Ser), c.2572T>A (p.Trp858Arg), c.550delA (p.Ile184Leufs^*^33), c.610insCCAA (p.Lys204Thrfs^*^26; individuals 12 and 21), c.1222C>T (p.Gly408^*^; individuals 24, 25 and 26) and c.2748C>G (p.Tyr916^*^) variants were consistently below the normal range. For C3bBbP, the three measurements in individual 18 carrying the c.1069T>A (p.Cys357Ser) variant was consistently above the normal range.

**Figure 4:**
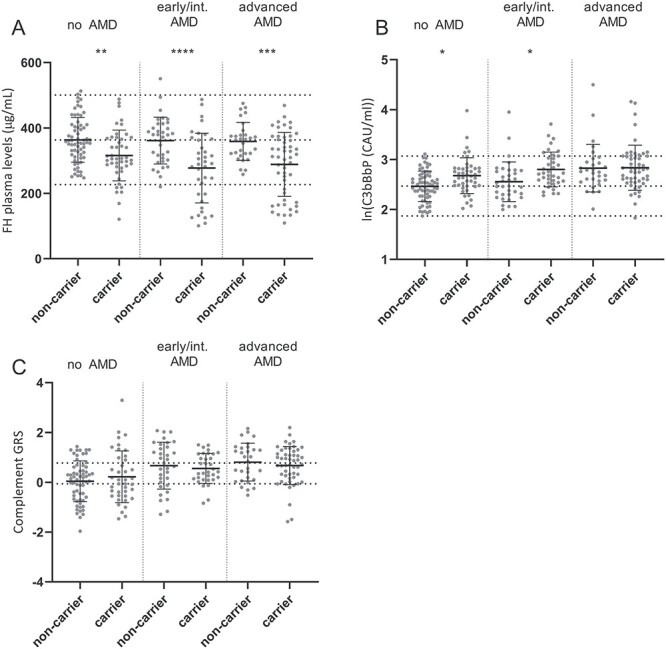
Differences in systemic complement levels and complement GRS in AMD patients and control individuals. (**A**) FH, (**B**) C3bBbP levels in plasma and (**C**) the complement GRS are shown for non-carriers and *CFH* rare variant carriers stratified by phenotype (no AMD, early/intermediate AMD and advanced AMD). (A–C) Mean and SD are shown by error bars, and (A, B) mean ± 2 SD is indicated by dotted lines. (C) Dotted lines indicate the tertiles of the complement GRS based on a large AMD case–control cohort (de Breuk *et al*., submitted for publication). Carriers and non-carriers within each AMD stage were compared with one-way ANOVA and Sidak’s multiple comparison test, and significant *P*-values are indicated with ^*^<0.05, ^*^^*^<0.01, ^*^^*^^*^<0.001 and ^*^^*^^*^^*^<0.0001.

### Systemic activation levels differ between AMD stages

It was previously reported that complement activation measured as C3d/C3 ratio increased with AMD progression (13). Here, with C3bBbP as a complement activation marker, the same direction of effect is observed. Therefore, we compared FH and C3bBbP levels of *CFH* rare variant carriers with non-carriers stratified by AMD stage. FH plasma levels were significantly decreased in carriers compared to non-carriers independent of AMD stage (Fig. 4A). For C3bBbP plasma levels, however, a significant increase was only observed in carriers without AMD or with early/intermediate AMD compared to non-carriers with the same disease stage. Strikingly, in advanced AMD patients, no difference in C3bBbP levels between carriers [mean 2.83 ln(CAU/ml)] and non-carriers [mean 2.84 ln(CAU/ml)] was observed (Fig. 4B).

The equally high C3bBbP levels in the carriers and non-carriers suggest that additional factors contribute to elevated C3bBbP complex formation in advanced AMD. Therefore, the complement genetic risk score (GRS) based on AMD-associated genetic variants in or near complement genes was calculated and compared between the groups. No difference in complement GRS was observed between the carrier and non-carrier groups. We could therefore not confirm that the complement GRS contributes to elevated C3bBbP complex formation in advanced AMD. To determine whether the complement GRS and C3bBbP levels are correlated, the Spearman correlation test was performed. A significant correlation was observed in non-carriers with early/intermediate AMD, where an increased complement GRS was associated with increased C3bBbP levels. However, no significant correlations were observed between GRS and C3bBbP levels in any of the other groups (Table 2).

**Table 2:** Correlation between complement GRS and C3bBbP levels

Group	*N*	Correlation coefficient^*^	*P*-value^*^
No AMD, non-carrier	61	0.216	0.094
No AMD, carrier	39	-0.029	0.861
Early/intermediate AMD, non-carrier	33	0.467^^*^^*^^	0.006
Early/intermediate AMD, carrier	33	-0.233	0.193
Late AMD, non-carrier	30	0.001	0.997
Late AMD, carrier	49	0.070	0.631

^*^Based on Spearman correlation test.

^*^^*^Correlation is significant at the 0.01 level (two-tailed).

### FH and C3bBbP plasma levels within families

**Figure 5:**
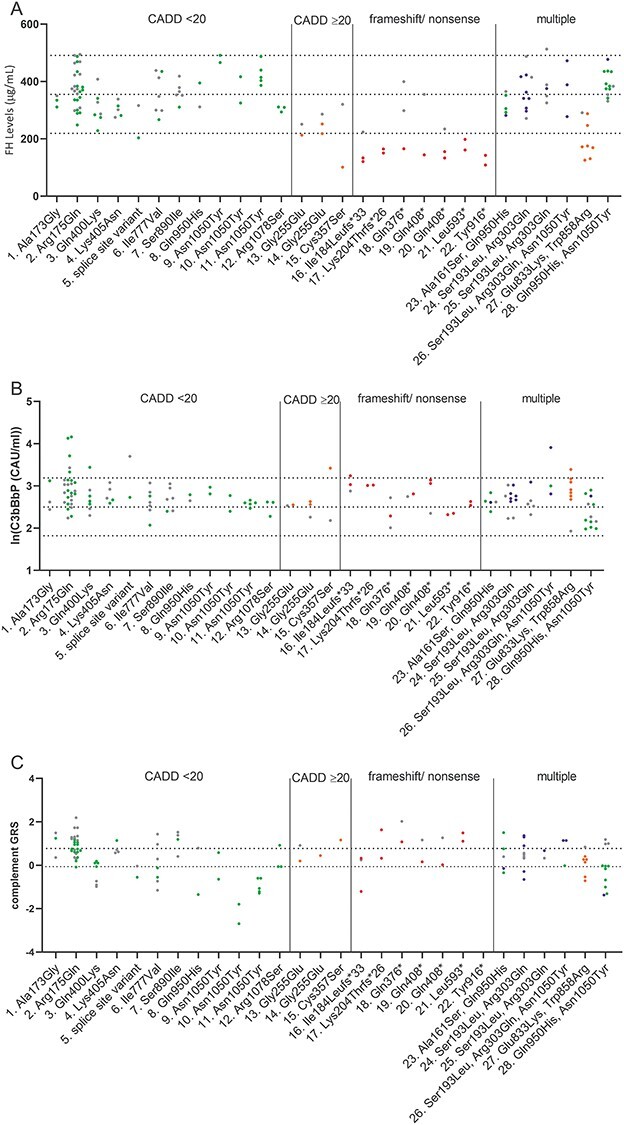
FH, C3bBbP plasma levels and complement GRS within families. (**A**) FH, (**B**) ln(C3bBbP) plasma levels and (**C**) the complement GRS are shown for each family. Individuals are color coded based on the *CFH* variant present: gray indicates non-carriers, green indicates individuals with a missense variant with CADD <20, oranges indicate individuals with a missense variant with CADD ≥20, red indicates individuals with a nonsense or frameshift variant and blue indicates individuals with multiple variants. (A, B) Dotted lines indicate the mean ± 2 SD based on the non-carrier controls shown in Figure 2. (C) Dotted lines indicate tertile ranges of the complement GRS as described in Figure 4. Mixed models analysis was performed to compare FH and C3bBbP plasma levels between carriers and non-carriers within the same family. Compared to non-carrier family members, individuals with a missense variant with CADD ≥20 or with a nonsense or frameshift variant show significantly reduced FH plasma levels. No significant differences in ln(C3bBbP) plasma levels were observed between carriers and non-carriers of the same family (Supplementary Material, Table S4).

Multiple genetic and environmental factors can influence complement activation, potentially masking the variants’ effects when measuring C3bBbP levels in plasma. We hypothesized that within families these factors might be more comparable. Therefore, we performed a sub-analysis in the family cohort and compared FH and C3bBbP plasma levels in rare and low-frequency *CFH* variant carriers with non-carriers by using mixed models analysis with family as a random factor. We categorized the families into those carrying rare *CFH* variants with CADD <20, CADD ≥20, frameshift or nonsense variants and multiple variants. Individuals carrying a variant with CADD ≥20 and individuals carrying a frameshift or nonsense variant showed significantly reduced FH plasma levels compared to non-carrier family members. No significant difference was observed for C3bBbP plasma levels in carriers compared to non-carrier family members. We then determined whether the complement GRS may contribute to C3bBbP levels in non-carrier family members. Family members carrying a variant with a CADD <20 presented with significantly lower complement GRS than non-carrier family members, but no difference in complement GRS was seen in carriers of other rare *CFH* variants compared to non-carrier family members (Fig. 5 and Supplementary Material, Table S4).

**Figure 6:**
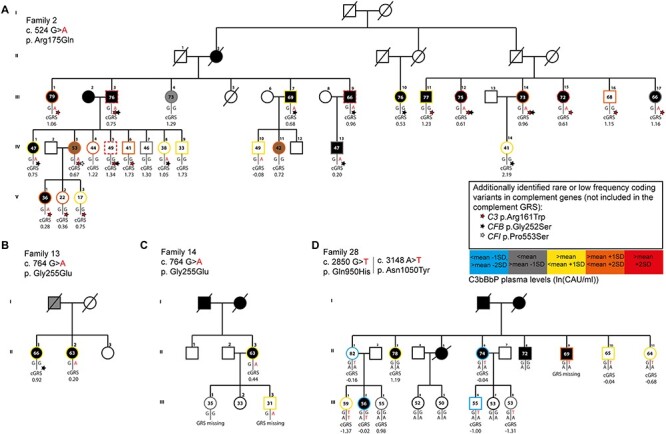
Pedigrees of selected families are shown. (**A**) In family 2, the variant c.524G>A (p.Arg175Gln) was identified in 14 of 27 individuals. (**B**) In family 13, the variant c.764G>A (p.Gly255Glu) was identified in one of two individuals. (**C**) In family 14, the variant c.764G>A (p.Gly255Glu) was identified in two of four individuals. (**D**) From family 28, 11 individuals were included in this study. The variant c.2850G>T (p.Gln950His) is present in eight individuals and the variant c.3148A>T (p.Asn1050Tyr) in two individuals. Individuals affected by AMD are indicated with a black symbol. The outer line color indicates ln(C3bBbP) levels. Ln(C3bBbP) levels above the mean + 2 SD (>3.2 ln(CAU/ml)) are shown with dashed lines in red, levels between the mean + 1SD and mean + 2SD (>2.85, <3.2 ln(CAU/ml)) are indicated in orange, levels between the mean and the mean + 1 SD (>2.51, <2.85 ln(CAU/ml)) are indicated in yellow, levels between the mean and the mean −1 SD (>2.17, <2.51 ln(CAU/ml)) are indicated in gray and levels between the mean −1 SD and the mean −2 SD (>1.83, <2.17 ln(CAU/ml)) are indicated in light blue. Family members not included in this study are indicated by a black outer line. The complement GRS is shown below each individual, and the presence of an additional coding variant not included in the GRS is indicated by a star (red = *C3* p.Arg161Trp, black = *CFB* p.Gly252Ser, white = *CFI* p.Pro553Ser). The age at sampling time point is indicated in years within each symbol.

To zoom further in on the family level, four selected families are depicted in Figure 6. Family 2 is a large family with 27 family members. Out of the 27 family members, 14 carry the *CFH* variant c.524G>A (p.Arg175Gln). This family presents with a high mean complement GRS of 0.918 (SD = 0.51) and with overall high C3bBbP plasma levels [mean = 2.96 ln(CAU/ml), SD = 0.48 ln(CAU/ml); non-carrier control range: 1.9–3.1 ln(CAU/ml)]. In this family, 14 individuals are also heterozygous carriers of the rare variant c.481C>T (p.Arg161Trp) in *C3* and 11 individuals also carry the low frequency variant c.724A>C (p.Gly252Ser) in complement factor B (*CFB*) (Fig. 6A).

In families 13 and 14, the rare *CFH* variant c.764G>A (p.Gly255Glu) was identified in one and two carriers, respectively (Fig. 6B and C). This variant was predicted to be damaging (CADD score ≥20) and showed significantly reduced FH levels in plasma (Fig. 2). Two out of the three carriers (family 13 individual II:2 and family 14 individual II:3) of this variant were diagnosed with AMD at a relatively young age (57 and 52 years, respectively). At the time of examination, both patients were already affected by advanced AMD at an age of 63 years. In both individuals, the complement GRS was intermediate (0.20 and 0.44, respectively), suggesting that their severe phenotype might be explained by the rare *CFH* variant. The third carrier (family 14 individual III:3) was a young family member who has not yet reached the age of potential AMD onset. The (complement) GRS in this family member was not available. In family 28, the benign variants c.2850G>T (p.Gln950His) and c.3148A>T (p.Asn1050Tyr) were identified in eight and two individuals out of the 15 family members, respectively. Plasma levels were measured in 11 individuals in this family, which presented with a low mean complement GRS of −0.13 and a mean C3bBbP plasma level of 2.33 ln(CAU/ml) [non-carrier control range: 1.9–3.1 ln(CAU/ml)] (Fig. 6D).

### C3bBbP levels in carriers of rare variants in other complement genes

In addition to a rare or low-frequency variant in *CFH*, 59 *CFH* variant carriers also carried a rare or low-frequency variant in *CFI* (4 individuals), *CFB* (38 individuals), *C3* (7 individuals) or variants in multiple of these genes (10 individuals). Of the family members without a rare or low-frequency variant in *CFH*, 2 carried a variant in *CFI*, 25 a variant in *CFB*, 6 a variant in *C3* and 4 carried a variant in multiple of these genes. In order to determine whether these variants affect C3bBbP levels in plasma, C3bBbP levels of individuals with a variant in any of these genes were compared to non-carrier controls (Supplementary Material, Fig. S3).

Of the 15 carriers with the *CFH* c.524G>A (p.Arg175Gln) variant included in this study, 13 carry an additional rare or low frequency variant in CFB or C3. Of these 13, 6 (40%) additionally carry the low-frequency *CFB* c.724A>C (p.Gly252Ser) variant, 3 (20%) the rare *C3* c.481C>T (p.Arg161Trp) variant and 4 (27%) carry both the *CFB* c.724A>C (p.Gly252Ser) and *C3* c.481C>T (p.Arg161Trp) variants. When stratifying carriers with the *CFH* c.524G>A (p.Arg175Gln) variant based on the presence of the additional *C3* and *CFB* variants, only carriers of all 3 variants [*CFH* c.524G>A (p.Arg175Gln), *C3* c.481C>T (p.Arg161Trp) and *CFB* c.724A>C (p.Gly252Ser)] showed significantly elevated C3bBbP plasma levels compared to controls. It should be noted, however, that there is a trend of increasing C3bBbP plasma levels for the different variant combinations according to the following order: *CFB* c.724A>C (p.Gly252Ser) (2.6 ln(CAU/ml)), *CFH* c.524G>A (p.Arg175Gln) (2.7 ln(CAU/ml)), *CFB* c.724A>C (p.Gly252Ser)/*CFH* c.524G>A (p.Arg175Gln) (2.8 ln(CAU/ml)), *C3* c.481C>T (p.Arg161Trp) (2.9 ln(CAU/ml)), *C3* c.481C>T (p.Arg161Trp)/*CFH* c.524G>A (p.Arg175Gln) (3.0 ln(CAU/ml)), *C3* c.481C>T (p.Arg161Trp)/*CFB* c.724A>C (p.Gly252Ser) (3.4 ln(CAU/ml)) (Supplementary Material, Fig. S3).

Of the nine carriers with the *CFH* variants c.578C>T;908G>A (p.Ser193Leu;Arg303Gln), and of the six carriers with the c.2572T>A (p.Trp858Arg) variant, one carrier each additionally carried the *CFB* variant c.724A>C (p.Gly252Ser). Since there is only one carrier for each combination available, no statistical test was performed with these individuals. However, the C3bBbP plasma levels of the carrier with the variants *CFH* c.578C>T;908G>A (p.Ser193Leu;Arg303Gln)/*CFB* c.724A>C (p.Gly252Ser) (2.8 ln(CAU/ml)) overlap with the range in carriers of only the *CFH* variants c.578C>T;908G>A (p.Ser193Leu;Arg303Gln) (2.6–3.9 ln(CAU/ml)). The C3bBbP plasma levels of the carrier with the variants *CFH* c.2572T>A (p.Trp858Arg) and *CFB* c.724A>C (p.Gly252Ser) are also within the same range of the C3bBbP plasma levels of *CFH* c.2572T>A (p.Trp858Arg) carriers (2.8 ln(CAU/ml) and 2.7–3.4 ln(CAU/ml), respectively).

## Discussion

In this study, we measured systemic FH and C3bBbP levels in plasma of 252 carriers of rare and low-frequency variants in *CFH* and in 159 non-carriers. In total, carriers of 64 different rare and low-frequency *CFH* variants were included in this study. For 8 variants, significantly reduced FH levels were identified in plasma of carriers, and for 11 variants, FH plasma levels were below the normal range at least in a single carrier (Fig. 2A). When stratifying individuals based on variant type, carriers of variants with a CADD <20, missense variants with a CADD ≥20 or nonsense or frameshift variants showed significantly reduced FH plasma levels compared to non-carrier cases and non-carrier controls (Fig. 2C). C3bBbP levels were significantly elevated in carriers of three *CFH* variants, and for nine variants, C3bBbP levels of a single carrier were above the normal range (Fig. 2B).

For 14 of the 64 variants included in this study, previous analyses either in plasma or serum or with purified protein have been reported (Supplementary Material, Table S1). For 10 variants, our results are in agreement with the previous findings, while for 3 variants our results differed. For the variant c.3148A>T (p.Asn1050Tyr), plasma levels were reported in combination with other *CFH* variants; therefore, no direct comparison is possible (14). For the variant c.481G>T (p.Ala161Ser), normal FH levels were reported (15–17), which is in line with our findings in two individuals. For the variant c.2669G>T (p.Ser890Ile), normal function was observed with purified protein (12). In accordance with this, five carriers of this variant (of which four carried the additional c.3019G>C (p.Val1007Leu) variant) present with normal FH and C3bBbP levels in plasma in this study. Carriers with the variant c.524G>A (p.Arg175Gln) present with normal FH levels and increased C3bBbP, which is in line with previous reports of impaired co-factor activity in serum of carriers (18). This variant is located in CCP3, which is part of the C3b binding site (19); therefore, impaired binding to C3b likely results in decreased co-factor activity.

For the variant c.578C>T (p.Ser193Leu), reduced co-factor activity in serum of carriers was observed in a previous study (18). In line with this, we observe that carriers of the combined variants c.578C>T;908G>A (p.Ser193Leu;Arg303Gln) have significantly elevated C3bBbP levels. For the variant c.172T>G (p.Ser58Ala), reduced FH plasma levels were reported in four heterozygous carriers using an allele-specific ELISA, and reduced activity in decay accelerating and co-factor functions were identified when testing purified protein (20). We observe normal FH and C3bBbP levels in a single individual carrying this variant. It should be noted, however, that in our study total FH and FHL-1 plasma levels were determined with ELISA, indicating that the effect of the variant is potentially masked by the *CFH* WT allele. Two individuals with the heterozygous variant c.1198C>A (p.Gln400Lys) presented with reduced FH plasma levels (21), while here we observe normal FH plasma levels in five individuals. It should be noted, however, that these individuals show FH plasma levels in the lower half and C3bBbP plasma levels in the upper half of the normal range, respectively.

For the truncating variant c.2141C>G (p.Ser714^*^), one carrier with normal FH plasma levels was reported (22), while in this study we observe reduced FH plasma levels in two carriers. Potentially the variants effect on FH plasma levels in the previous report was masked by interindividual variation in the WT allele.

Furthermore, 25 individuals with multiple *CFH* variants were included in this study. One individual carrying the variants c.211T>A(;)3148A>T (p.Trp71Arg(;)Asn1050Tyr) presented with low FH and high C3bBbP plasma levels and one individual carrying the variants c.3130A>G(;)3148A>T (p.Arg1044Gly(;)Asn1050Tyr) presented with low FH plasma levels. Since carriers with only the variant c.3148A>T (p.Asn1050Tyr) showed normal FH and C3bBbP plasma levels, the observed effect is most likely due to the c.211A>T (p.Trp71Arg) and c.3130A>G (p.Arg1044Gly) variants, respectively (Fig. 2A and B).

For 35 (55%) of the 64 variants that were included in this study, no significant difference or clustering of FH or C3bBbP plasma levels outside the normal range was identified. These variants may either be benign, may have a mild effect that is masked by the WT allele or they may have an effect on other functions of FH that were not assessed in this study. Here, we measured C3bBbP plasma levels as a systemic activation marker for complement activation. Regulation of inflammatory processes locally at the tissue level, e.g. RPE, and binding properties of FH to extracellular components like glycosaminoglycans that are characteristic of host cells, are not evaluated. Additional studies using binding assays to, e.g., glycosaminoglycans are needed to determine the significance of these variants locally at the tissue of interest.

The 64 variants included in this study are distributed throughout the entire FH protein, with 31 (48%) variants also being present in FHL-1; consequently, these variants potentially affect both proteins (Fig. 1B). In AMD patients, a burden of rare coding variants in the CCP domains one to four has been identified (19), and while FHL-1 is with 10–50 μg/ml less abundant than full-length FH in plasma (23), equal concentrations of FHL-1 and FH have been identified in aqueous humor likely due to higher permeability of Bruch’s membrane for FHL-1 (7,24). While these findings point to a special role of FHL-1 in the context of ocular complement regulation, also variants outside of FHL-1, like the c.3628C>T (p.Arg1210Cys) variant, were shown to be associated with increased AMD risk (5,10), indicating that reduced availability of full-length FH is also a risk factor for AMD. Here, we observe significantly reduced FH plasma levels for the truncating variants c.1778T>A (p.Leu593^*^), c.2141C>G (p.Ser714^*^) and c.2748C>G (p.Tyr916^*^) and for the missense variant c.2572T>A (p.Trp858Arg). Since these variants are located in CCP10, CCP12, CCP15 and CCP14, respectively, FHL-1 secretion is potentially not affected by these variants.

The common variant c.1204T>C (p.Tyr402His) is located in CCP7 and affects the binding capacities of both FH and FHL-1 (24). In carriers of *CFH* variants in CCP8–20, more emphasis on the remaining FHL-1 protein might be given; therefore, the allele frequency of the variant rs570618, which is in perfect LD with c.1204T>C (p.Tyr402His), was analyzed. The protective variants c.2850G>T (p.Gln950His) and c.3148A>T (p.Asn1050Tyr) are in LD with the protective allele of the c.1204T>C (p.Tyr402His) variant; in line with this, when including carriers with these two variants, the protective allele G of rs570618 is enriched in carriers of a *CFH* variant in CCP8–20, but the frequencies normalize when excluding the carriers of c.2850G>T (p.Gln950His) and c.3148A>T (p.Asn1050Tyr) (Supplementary Material, Table S2).

When splitting *CFH* variant carriers based on whether the variant is located in CCP1–7 or in CCP8–20, equally reduced FH plasma levels are observed in both groups, but carriers of *CFH* variants in CCP1–7 showed significantly elevated C3bBbP plasma levels (Supplementary Material, Fig. S2). This observation is in line with CCP1–4 being necessary for the decay accelerating activity and co-factor function of FH (25). Here, we measured systemic C3bBbP levels as a marker of complement activation; however, one could hypothesize that variants affecting FHL-1, which was previously shown to be present in the retina (7), have a more severe local effect in the retina compared to variants only affecting full-length FH. In risk allele carriers of the common variant c.1204T>C (p.Tyr402His), increased levels of C5b-9 and C-reactive protein in the choroid (26,27) and reduced choroidal thickness were reported (28) pointing towards local effects. It seems likely that rare and low-frequency variants also have comparable or maybe even more severe effects locally, but this will need to be confirmed in future studies.

When grouping individuals based on variant type, carriers of missense, nonsense or frameshift variants showed significantly reduced FH levels compared to non-carrier controls and non-carrier cases (Fig. 2C). Additionally, carriers of missense and nonsense or frameshift variants showed significantly elevated C3bBbP levels compared to non-carrier controls but not to non-carrier cases (Fig. 2D). In line with previous reports showing elevated systemic complement activation in AMD patients (29–33), non-carrier AMD cases show significantly elevated C3bBbP levels compared to non-carrier controls (Fig. 2D).

Measurement of C3bBbP plasma levels was previously used as a biomarker to determine systemic complement activation in patients with atypical hemolytic uremic syndrome (34), in patients with myocardial infarction (35) and recently in patients with respiratory failure due to COVID-19 (36). Here, we report C3bBbP plasma levels as a biomarker for complement activation in AMD patients for the first time. Measurement of C3bBbP plasma levels in follow-up samples showed that it is stable over time in most patients, except for seven (24%) cases (individuals 1, 12, 19, 20, 24, 25 and 26) (Fig. 3). It should be noted, however, that no information on recent infection or other co-morbidities was collected during the patients’ visits, while such factors could lead to short-term alterations in complement activation.

Recently, it was reported that systemic and local complement activation levels are related to disease stage in AMD (13,37). Therefore, we compared systemic complement levels between *CFH* rare variant carriers and non-carriers stratified by AMD stage. In all three disease stages (no AMD, early/intermediate AMD and advanced AMD), rare variant carriers had significantly lower FH plasma levels compared to non-carriers (Fig. 4A). Interestingly, while *CFH* rare variant carriers without AMD or with early/intermediate AMD showed significantly elevated C3bBbP levels compared to non-carriers, no such difference was observed between carriers and non-carriers with advanced AMD (Fig. 4D). We previously reported that advanced AMD patients have a higher GRS based on all 52 AMD-associated variants (38); therefore, we hypothesized that non-carriers with late AMD might carry a higher burden of AMD-associated variants in complement components. However, when comparing the complement GRS between carriers and non-carriers for all groups, no significant differences were observed (Fig. 4C). In advanced AMD patients, the complement GRS was equally high in carriers and non-carriers of rare *CFH* variants. It was previously shown that patients with advanced AMD have a higher complement GRS than individuals without AMD or with early/intermediate AMD (39). This further supports that genetic variants assessed in the complement GRS could contribute to elevated C3bBbP complex formation in advanced AMD. Though we did not detect a significant correlation between C3bBbP levels and complement GRS in non-carrier advanced AMD cases, a correlation was observed in non-carrier early/intermediate AMD cases. This might suggest that additional factors to complement activation contribute to disease pathogenesis in advanced AMD patients. It should be noted that the groups included in the correlation analysis are quite small and therefore the correlation analysis needs to be confirmed in a larger cohort. It has been speculated that the active macular disease process elicits a local inflammatory signal, especially in those with a high genetic burden, resulting in a self-perpetuating amplification loop of increasing complement activation which also affects systemic complement levels (13,40).

When examining the variants on a familial level, carriers of variants with CADD ≥20 and of nonsense or frameshift variants showed significantly decreased FH plasma levels compared to non-carrier family members, but no significant difference in C3bBbP plasma levels between carriers and non-carriers was observed (Fig. 5). However, the sizes of families differ, and especially, the seven families with nonsense variants are relatively small with on average 2.4 family members.

Family 2 presents with an overall high complement activation. In addition to the c.524A>G (p.Arg175Gln) variant in *CFH*, the rare variant c.481C>T (p.Arg161Trp) in *C3* was identified in 14 family members, the rare variant c.1657C>T (p.Pro553Ser) in *CFI* was identified in one family member and the low-frequency variant c.742A>C (p.Gly252Ser) in *CFB* was identified in 11 family members (Fig. 6A). For the *CFI* variant c.1657C>T (p.Pro553Ser), normal FI secretion, but a mild impairment in C3b degradation in plasma was reported (18,41,42), indicating that the variant potentially has a mild influence on the C3bBbP plasma levels in the carrier of family 2. For the *C3* variant c.481C>T (p.Arg161Trp), normal C3 plasma levels but decreased binding of FH were reported (18,43), and for the *CFB* variant c.742A>C (p.Gly252Ser), no functional data are available to date. This variant is located in the Ba fragment of FB and thus not part of the C3bBbP complex (Fig. 1A). Interestingly, while individuals carrying one or two of the variants *CFH* c.524G>A (p.Arg175Gln), *C3* c.481C>T (p.Arg161Trp) or *CFB* c.724A>C (p.Gly252Ser) did not show significantly elevated C3bBbP plasma levels, carriers of all three variants show significantly elevated C3bBbP levels compared to non-carrier controls (Supplementary Material, Fig. S2). Since both *CFH* c.524G>A (p.Arg175Gln) and *C3* c.481C>T (p.Arg161Trp) potentially affect binding of FH to C3b (19,43), our findings indicate an additive detrimental effect of these variants.

Family 28 presents with low complement GRS, and C3bBbP plasma levels within the non-carrier control range (Fig. 6D). This family, however, carries high risk genotypes at the ARMS2/HTRA1 locus, indicating that the complement system may not be a main driver in the AMD affected individuals from this family. This may be of relevance for currently ongoing clinical trials for complement inhibition therapy, as patients with a low complement GRS and low C3bBbP levels might not benefit from such new treatment approaches.

Considering the important role of the complement pathway in AMD, multiple ongoing clinical trials are focusing on inhibition of the complement system, and some even include patients in these trials based on genotype. One of the trials selecting patients based on genotype is GEM103 (NCT04246866), which is based on the intravitreal supplementation of human FH protein. AMD patients carrying mutations in *CFH* that result in reduced FH levels or impaired FH function might benefit most from this ongoing trial, which aims to restore complement regulation. If complement regulating trials are successful, identification of AMD patients carrying rare *CFH* variants will become important. Although genetic testing is not a standard procedure in AMD care to date, it may be considered for specific subgroups, who have a higher chance to carry mutations in *CFH*, such as families with a high prevalence of AMD (De Breuk *et al*., submitted for publication), or AMD patients with specific phenotypic fundus characteristics (44), or patients with AMD characteristics at an early age (de Breuk *et al*., under review). Functional testing to determine whether specific rare *CFH* variants affect FH levels or function, as shown in this current study, will be helpful in this context.

In conclusion, rare variants in the *CFH* gene can lead to reduced FH levels or reduced FH function as measured by increased C3bBbP levels. We identified reduced FH plasma levels in carriers of eight variants and elevated C3bBbP plasma levels in carriers of three variants. Eight variants identified in a single carrier showed FH plasma levels below the normal range and nine variants identified in a single carrier showed C3bBbP plasma levels above the normal range, pointing towards these variants being pathogenic. In light of natural variation, however, these findings need to be confirmed either in plasma from additional carriers or with recombinantly expressed protein. The described effects of individual variants in the *CFH* gene reported in this study will improve the interpretation of rare and low-frequency variants observed in AMD patients in clinical practice and will aid in the selection of patients for clinical trials.

## Materials and Methods

### Study population

Individuals from the European Genetic Database were included based on genotype, AMD grading and family status. All enrolled participants gave written informed consent and were pseudo-anonymized with a database identifier code. The study was approved by the local ethics committees on Research Involving Human Subjects and conducted according to the Declaration of Helsinki.

The total study population consisted of 411 individuals, divided into two sub-cohorts: an unrelated case–control cohort (273 individuals) and a family cohort (138 individuals from 28 families with at least one AMD affected individual). In the unrelated case–control cohort, we included 168 carriers of rare or low-frequency *CFH* variants (85 no AMD, 38 early or intermediate AMD, 45 advanced AMD) and 105 *CFH* non-carriers (53 no AMD, 25 early or intermediate AMD, 27 advanced AMD). The family cohort consisted of 84 family members carrying rare or low-frequency variants in the *CFH* gene (35 no AMD, 25 early or intermediate AMD, 24 advanced AMD) and 54 *CFH* non-carrier family members (41 no AMD, 10 early or intermediate AMD, 3 advanced AMD) (Table 1). For 29 individuals, plasma samples from follow-up visits were included in this study (Supplementary Material, Table S2).

AMD diagnosis was obtained by evaluation of retinal images according to the Cologne Image Reading Center and Laboratory (CIRCL) protocol (45). In 16% of the individuals, CIRCL grading was not available, and retinal images were graded according to the Wisconsin age-related maculopathy grading system and reclassified into the Rotterdam Classification (RC) (46–48). We assigned all individuals to one of the three phenotype groups: no AMD [no signs of AMD or ≤10 small drusen ≤63 μm together with pigmentary changes (CIRCL); RC grade 0], early/intermediate AMD [≥10 small drusen ≤63 μm together with pigmentary changes or ≥1 intermediate drusen of 63–124 μm or large drusen ≥125 μm (CIRCL); RC grade 1–3] or advanced AMD [choroidal neovascularization or geographic atrophy in at least one eye (CIRCL); RC grade 4].

EDTA plasma samples were collected and centrifuged according to standard protocols and frozen at −80°C within 1 h. The samples were stored at −80°C at the Ophthalmology Department of the Radboudumc or in the Radboud Biobank (49). Genomic DNA isolation from peripheral blood was performed according to standard procedures.

### Genotyping

Genotyping data based on whole exome sequencing (50), single-molecule molecular inversion probes in combination with next generation sequencing (38) or a customized HumanCoreExome array (5) were available for the individuals included in this study. All genotyping passed quality control as described in the original studies (5,38,50). In a small number of individuals, a panel of complement genes (*C3, CFB, CFH, CFI*) was sequenced in clinical diagnostic setting at the Laboratory for Diagnostics of the Radboudumc using Sanger sequencing or next generation sequencing (Illumina NextSeq 500). We filtered for carriers of rare and low-frequency variants [minor allele frequency <0.01 and between 0.01 and 0.05, respectively, based on the non-Finnish European population (gnomAD)] in the *CFH* gene, referred to as *CFH* carriers. Individuals without rare or low-frequency variants in the *CFH* gene were labeled as *CFH* non-carriers. Considering the potential influence of rare and low-frequency variants in the other complement genes, we additionally filtered for carriers of rare and low-frequency variants in the *CFI, CFB* and *C3* genes in the case–control cohort. All identified *CFH* variants were confirmed by Sanger sequencing, unless they were identified on more than one genotyping platform. *CFH* variants c.2850G>T (p.Gln950His) and c.3148A>T (p.Asn1050Tyr) were not further confirmed since they were found relatively frequent. *CFH* variants are annotated based on NM_000186.4 and GRCh37 (hg19) build, and the protein numbering is indicated including the signal peptide. CADD scores for the identified *CFH* variants were calculated with the annotation pipeline of the Human Genetics Department of the Radboudumc and categorized into four groups: (1) CADD <20, (2) CADD ≥20, (3) nonsense or frameshift variants and (4) multiple *CFH* variants.

We extracted all 19 complement variants of the 52 AMD-associated variants, as reported in the study of the International Age-related Macular Degeneration Genomics Consortium (IAMDGC) (5) from the single-molecule molecular inversion probes and customized HumanCoreExome array datasets and calculated a complement GRS for each individual from whom the four major risk and protective complement variants (*CFH* rs570618, *CFH* rs10922109, *C2/CFB/SKIV2L* rs429608 and *C3* rs2230199) were available. We used the following formula: $GRS={\sum}_{i=1}^{52}\Big({G}_i{\beta}_i\Big)$, where ${G}_i$ represents the genotype of variant $i$ (coded as 0, 1 or 2 based on the number of minor alleles) and ${\beta}_i$ represents the effect size of variant $i$ (natural logarithm of the fully conditioned odds ratio of the minor allele of variant $i$, based on the study of the IAMDGC) (5). For 4/19 complement variants within the single-molecule molecular inversion probes dataset, an alternative single-nucleotide polymorphism in high LD was used, since the original variant was poorly covered (Supplementary Material, Table S5).

### FH and C3bBbP ELISA

Concentrations of native FH/FHL-1 and C3bBbP/C3bBbPC3b in EDTA plasma were determined using sandwich enzyme-linked immunosorbent assay (ELISA). High binding microplates (Greiner, Austria) were coated with 1000× diluted mouse anti-human Factor P#2, binding properdin (Quidel, USA) or with 10 000× diluted goat anti-human FH (Quidel, USA) in coating buffer (15 mm Na2CO3^*^10 H2O, 35 mm NaHCO3, pH 9.6). Plates were blocked with 1% bovine serum albumin (BSA, Sigma, USA) in PBS. The plates were washed four times with 200 μl PBS 0.02% Tween after blocking and between all the following incubation steps. EDTA plasma samples for the C3bBbP ELISA were diluted 10× in PBS-Tween 10 mm EDTA and incubated for 1 h at 4°C. As standard the international complement standard #2 was used (51). EDTA plasma samples for the FH ELISA were diluted 5000× in PBS with 0.02% Tween and 0.2% BSA. Serum-purified FH was used as standard (CompTech, USA). After applying the samples, C3bBbP complexes were detected with 2000× diluted rabbit anti-human C3c (Siemens, Germany), and FH/FHL-1 was detected with 10 000× diluted mouse anti-human FH (Abcam, UK). The secondary antibodies were horseradish peroxidase labeled Igs. For C3bBbP 2000× diluted goat anti-rabbit (Dako, Germany), and for FH goat anti-mouse (Dako, Germany) was applied, followed by detection with o-phenylenediamine dihydrochloride substrate (Dako, Germany), which was diluted according to manufacturer’s instructions. Each sample was measured in duplicate.

### Statistical analysis

Statistical analysis and data visualization were performed with IBM SPSS Statistics 25 (IBM Corp. Released 2017. IBM SPSS Statistics for Windows, Version 25.0. IBM Corp., Armonk, NY) and GraphPad Prism version 8.4.3 for Windows, GraphPad Software, San Diego, CA, www.graphpad.com. Natural log transformation was used to normalize the distribution of C3bBbP measurements, and all further analyses were performed with the transformed values. FH and C3bBbP plasma levels were compared with non-carrier controls using one-way analysis of variance (ANOVA) and Dunnett’s *post hoc* test or selected groups were compared with Sidak’s multiple comparison test. The correlation between the complement GRS and C3bBbP plasma levels was tested with Spearman correlation. It should be noted that for the non-carrier control group, family members below the age of 65 years were excluded. To compare FH and C3bBbP plasma levels between carriers and non-carriers within the same family, mixed models analysis was performed. For the variant rs570618, the allele frequencies were analyzed in non-carriers, carriers of a variant in CCP1–7 and carriers of a variant in CCP8–20 using Chi-squared test.

## Funding

Dutch Research Council (016.Vici.170.024 to A.I.d.H.), and a collaborative research agreement with Gemini Therapeutics, Inc.

*Conflict of Interest statement*. S.F. is an employee of F. Hoffmann-La Roche. S.K. is an employee of Gemini Therapeutics. A.I.d.H. is a consultant for Ionis Pharmaceuticals, Gyroscope Therapeutics, Gemini Therapeutics and F. Hoffmann-La Roche.

## Supplementary Material

supp_figure_1_ddab256

supp_figure_2_ddab256

supp_figure_3_ddab256

supp_table_1_ddab256

supp_table_2_ddab256
