# Supplementary figures and images for: Systemic complement levels in patients with age-related macular degeneration carrying rare or low-frequency variants in the CFH gene
Source: Hum Mol Genet. 2021 Sep 11;31(3):455–70. doi: 10.1093/hmg/ddab256 (PMC8825240; doi:10.1093/hmg/ddab256)

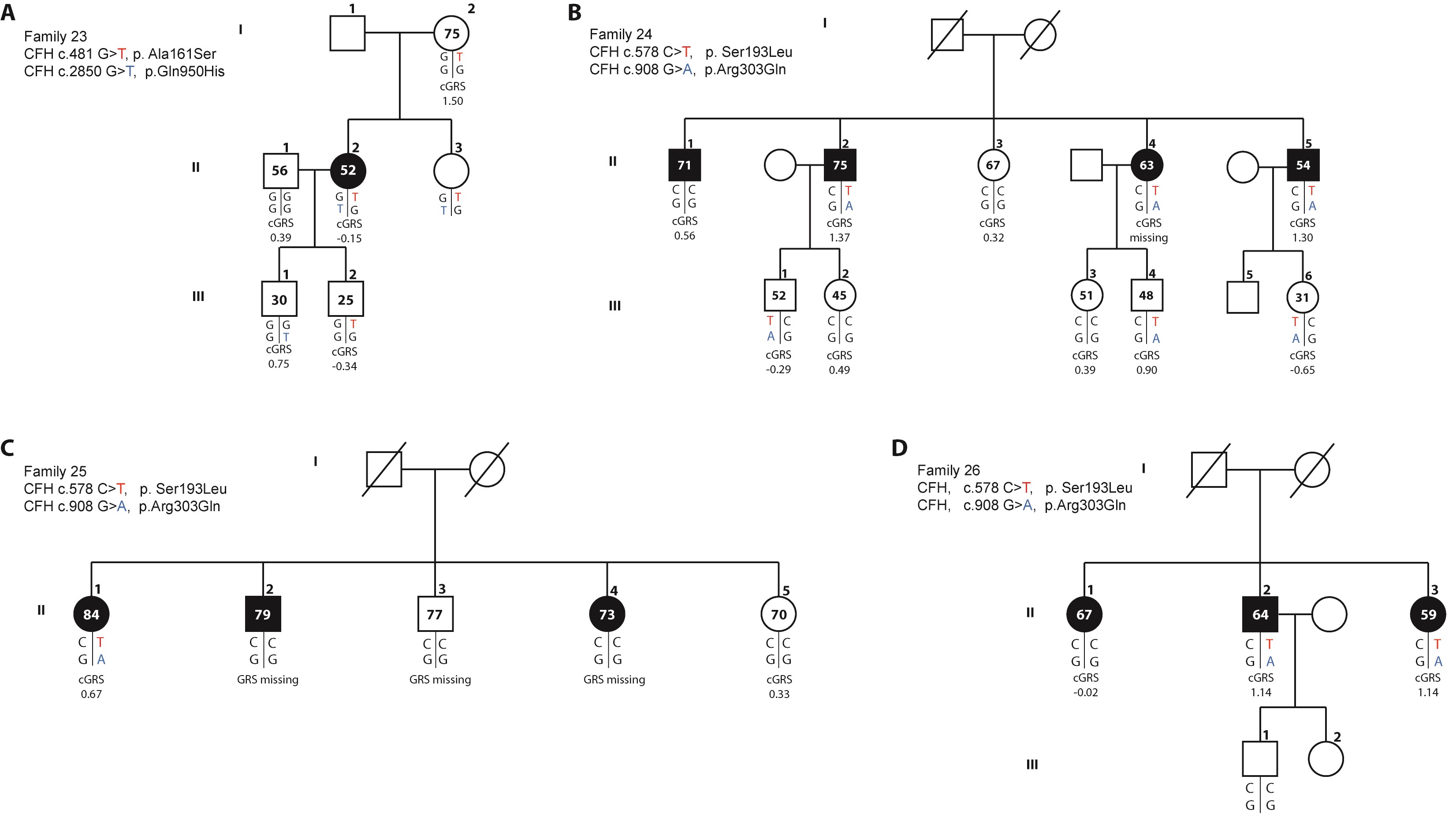

Supplement: supp_figure_1_ddab256 [file supp_figure_1_ddab256.jpeg]

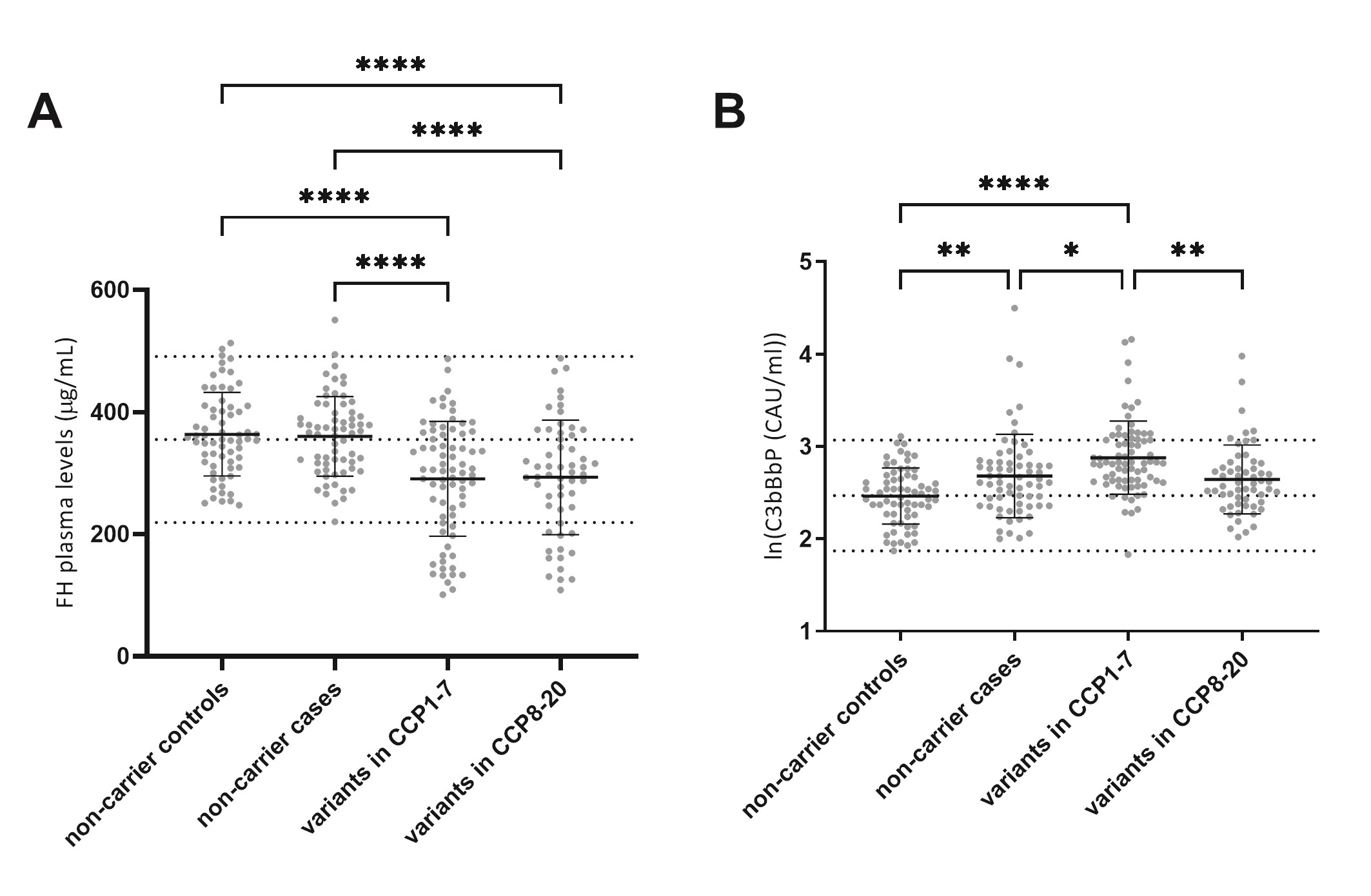

Supplement: supp_figure_2_ddab256 [file supp_figure_2_ddab256.jpeg]

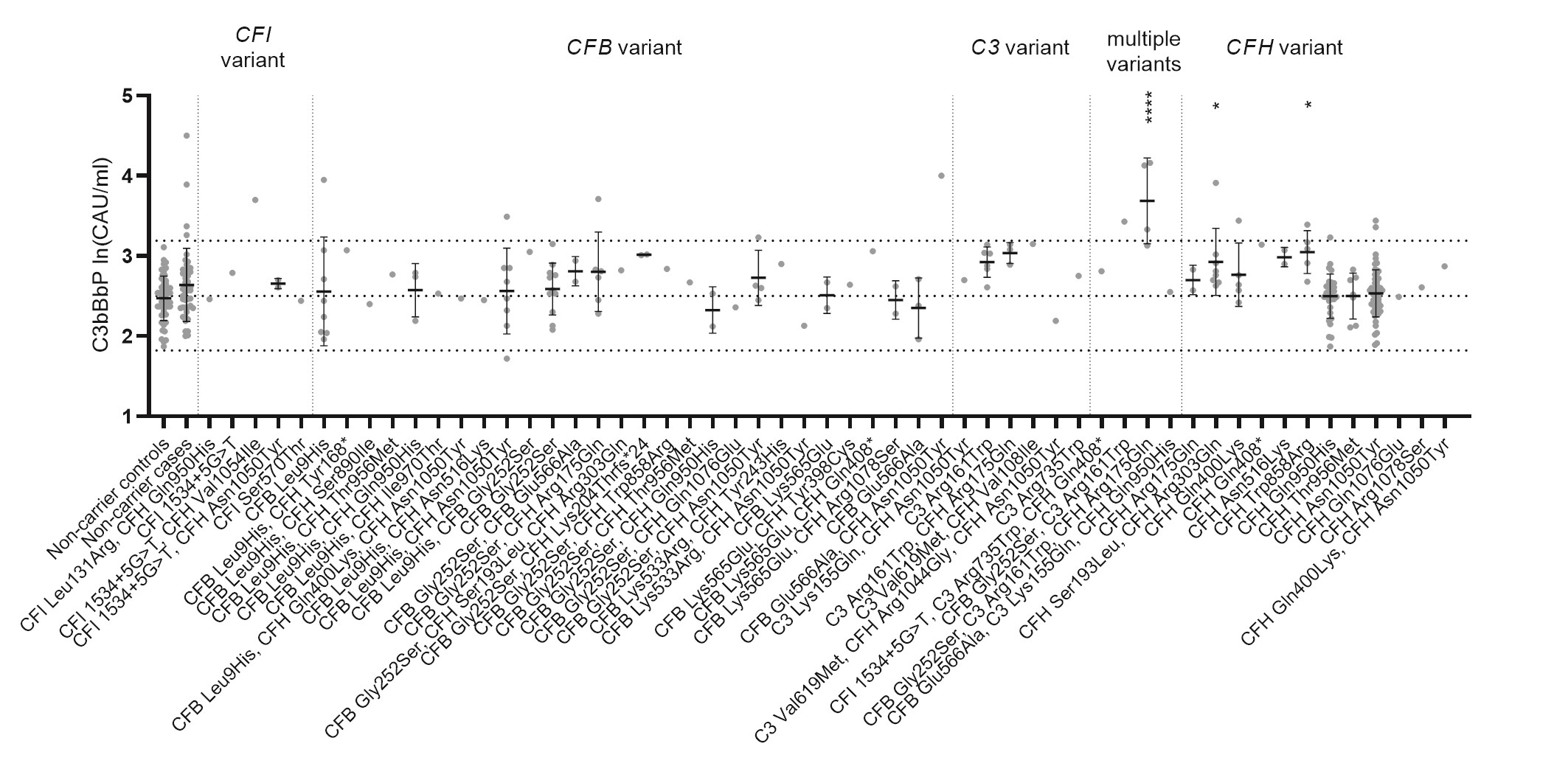

Supplement: supp_figure_3_ddab256 [file supp_figure_3_ddab256.jpeg]
